# A short delirium caregiver questionnaire for triage of elderly outpatients with cognitive impairment: a development and test accuracy study

**DOI:** 10.1017/S1041610219001595

**Published:** 2021-01

**Authors:** Hendrika J. Luijendijk, Daisy W. P. Quispel-Aggenbach, Anne J. M. Stroomer-van Wijk, Agnes H. Meijerink-Blom, Annemiek van Walbeek, Sytse U. Zuidema

**Affiliations:** 1 Department of General Practice and Elderly Care Medicine, University of Groningen, University Medical Center Groningen, Groningen, The Netherlands; 2 Department of Geriatric Psychiatry, Parnassia Psychiatric Institute, Rotterdam, The Netherlands; 3 Department of Geriatric Psychiatry, Parnassia Psychiatric Institute, The Hague, The Netherlands; 4 Department of Adult Psychiatry, Dimence Mental Health Care, Deventer, The Netherlands; 5 Department of Internal Medicine, Erasmus MC University Medical Centre Rotterdam, Rotterdam, The Netherlands

**Keywords:** delirium, screening, triage, caregiver, questionnaire, instrument, diagnosis, telephone

## Abstract

**Objectives:**

Delirium is often missed in older outpatients. Caregivers can give valuable information that might improve identification rates. The aim of this study was to develop a short and sensitive delirium caregiver questionnaire (DCQ) for triage of elderly outpatients with cognitive impairment by telephone.

**Design, setting, and participants:**

The pilot questionnaire was administered to 112 caregivers of patients who were referred for dementia screening to our clinic for geriatric psychiatry, and the final DCQ to 234 other caregivers.

**Measurements:**

In phase I (2013–2014), we tested a pilot questionnaire with 17 items. Health professionals who established delirium diagnoses were blinded to the results. We then used the results and other information available at referral to construct the final DCQ with seven items. During phase II (2015–2016), we investigated the test accuracy of the final DCQ in a subsequent cohort. In both phases, the patients received a structured diagnostic workup. Time between referral and first visit was a secondary outcome.

**Results:**

The final DCQ consisted of the following items: emergency visit required, sleeping disorder, fluctuating course, hallucinations, suspicious thoughts, previous delirium, and recent discharge from hospital. DCQ results indicated that urgent intake was required in 85 of 234 patients. Sensitivity was 73.5% (95% CI: 58.9–85.1%) and specificity 73.5% (95% CI: 66.5–79.7%). The mean number of days to first visit dropped from 31.6 to 11.2 in delirious patients (*p* = 0.001).

**Conclusions:**

Triage with the easy-to-use DCQ among patients referred for cognitive screening leads to earlier assessment and higher detection rates of delirium.

## Summary

Existing delirium screening tools are not suitable for triage in outpatients with dementia. We developed the short seven-item delirium caregiver questionnaire. The triage tool can be administered by telephone to caregivers. Its use leads to earlier assessment and higher detection rates of delirium.

## Introduction

Delirium is a serious and potentially debilitating neuropsychiatric syndrome. It is characterized by attention deficits, cognitive dysfunction, and neuropsychiatric symptoms that begin acutely and fluctuate throughout the day. Delirium affects 10–40% of hospitalized patients, 11–39% of nursing home patients, and 16–19% of elderly outpatients referred for cognitive screening to psychiatric clinics (De Lange *et al*., 2013; Hasegawa *et al*., 2013; Siddiqi *et al*., 2006; Stroomer-van Wijk *et al*., 2016). Cognitive impairment is an important risk factor for developing delirium (Hasegawa *et al*., 2013; Kazmierski *et al*., 2014; Oldham *et al*., 2015). It is important to distinguish between dementia with neuropsychiatric symptoms and dementia with delirium. The treatment of the latter is focused on the underlying somatic factors.

However, delirium can be difficult to identify. It is missed in 40–60% of hospitalized and nursing home patients (De Lange *et al*., 2013; Siddiqi *et al*., 2006). It is likely to be missed in outpatients of memory clinics as well because delirium is thought to occur only in very ill patients. Moreover, detecting delirium in patients with pre-existent dementia can be difficult compared to delirium in patients without pre-existent cognitive disorders. In patients with dementia, cognitive disorders – one of the core symptoms of delirium – have been present for some time before delirium occurs. Other symptoms such as paranoia, hallucinations, and wake–sleep disruptions may overlap as well.

Successful treatment of delirium depends on timely and adequate detection. To improve delirium detection rates, a number of screening instruments have been developed. Nurses can administer instruments such as the Delirium Observation Screening Scale (DOSS) and NEECHAM confusion scale (Neelon *et al*., 1996; Schuurmans *et al*., 2003). In addition, diagnostic tests such as the Confusion Assessment Method (CAM) have become popular (Inouye *et al*., 1990). As these tests require a lot of time to administer, they are not very suitable for triage in outpatient settings (Marcantonio *et al*., 1998). Moreover, they are not very sensitive to identifying delirium in patients with dementia. Short sensitive tests that can distinguish between dementia and delirium, such as the Observation Scale of Level of Arousal (OSLA) or Richmond Agitation Sedation Scale (RASS), rely on direct observation of the patients (Quispel-Aggenbach *et al*., 2018; Sessler *et al*., 2002; Tieges *et al*., 2013).

Caregivers know patients best and can observe (sub) acute changes in behavior well (Yevchak, 2013). We strived for a short and sensitive tool that could be administered by phone to obtain this information. Use of such an instrument for triage might not only increase detection rates but also reduce the time to identification. The aim of this study was to develop a delirium caregiver questionnaire (DCQ) for triage in older outpatients referred for cognitive screening.

## Methods

### Setting and patients

The current study consisted of two phases. In the first phase, we tested an extended pilot questionnaire to develop the short final DCQ. This study was performed as part of a larger cohort study to assess the prevalence of delirium among older patients consecutively referred for dementia screening to an outpatient clinic of a mental healthcare institution (Quispel-Aggenbach *et al*., 2018). In the second phase, we tested the diagnostic accuracy of the final DCQ in daily practice. The Medical Ethics Committee of the Erasmus University of Rotterdam, the Netherlands, approved the protocol of the cohort study, in which the current study was embedded.

Patients resided in or nearby Rotterdam, the Netherlands, and were referred by their general practitioner or a geriatrician. The referral reasons were dementia screening, or cognitive disorders with psychological or behavioural disorders that required counselling or treatment. The exclusion criteria were the request for consultation about a hospitalized or institutionalized patient, and second referral during study period.

### Study phases

During phase I, we examined which information that might suggest the presence of delirium could be solicited from caregivers. We composed a questionnaire with 17 items, including symptoms of delirium that a caregiver could have observed (see online appendix). It started with an open question about the referral reason (clinical need) and type of symptoms. This was followed by two questions about a (sub) acute onset, three questions about the presence of a fluctuating course, two about attention disorders, one about motoric unrest, one about motoric slowing, one about destructive behavior, one about chaotic behavior, one about sleep disorders, one about health changes, one about medication changes, and two about caregiver burden. We used multiple questions for some symptoms to find the most comprehensible wording. Each item could be scored as yes, no, or unclear.

We administered the questionnaire to caregivers of consecutively referred patients by telephone. One of three investigators (HJL, DWPQ, AHMB) approached them shortly after the referral. Health professionals responsible for delirium assessment (see below) were blinded to the results. To compose the short final DCQ, we first selected the items of the pilot questionnaire that were most sensitive to the presence of delirium. Then, we added items that might increase specificity and thus reduce the number of false-positive cases (see statistical analysis).

In phase II of this study, we introduced DCQ in two memory clinics of our institution. The person telephoning the caregiver to make the appointment for the first visit was to administer DCQ. This person was a secretary in the memory clinic in Rotterdam South, and a psychiatric nurse in the clinic in Rotterdam North. This nurse also performed delirium assessment (see below).

The primary outcome was test accuracy of DCQ. A secondary outcome was whether the introduction of DCQ would reduce time between referral and first assessment visit. Therefore, we registered the date of referral and date of first visit in phase I (usual care) and phase II (with DCQ).

### Delirium assessment

During phases I and II, patients received a psychiatric assessment by a registered psychiatric nurse and a geriatrician. At least two visits were made in case of regular referrals, usually at the patient’s home in the presence of the caregiver. If the patient lived in a home for the elderly (but not a nursing home), a nurse assistant was usually present. In case of a referral for an urgent visit, patients were mostly seen once but concurrently by a nurse and physician.

Patients, family, and nurse assistants were interviewed about the nature, severity, onset, and course of current and recent symptoms. This would include cognitive impairments and neuropsychiatric symptoms. In addition, the patient received a psychiatric examination. Cognitive functioning was tested with the mini-mental state examination and the clock drawing test (Bryson *et al*., 2011; Folstein *et al*., 1975)

During phase I, the Delirium Rating Scale-revised-98 (DRS-R-98) was used to support the systematic assessment of delirium (Trzepacz *et al*., 2001). The first 13 items cover the nature and severity of psychiatric symptoms and can be scored from 0 to 3. The last three diagnostic items refer to the onset, course, and somatic causes of delirium. Although the use of DRS-R-98 was not compulsory anymore during phase II, the rate of identified probable delirium was comparable to that in phase I (22% in phase I and 19% in phase II).

In addition to psychiatric assessment, a somatic examination was performed in all patients. We took a somatic history, performed a physical examination, and ordered standard blood and urine tests. We also recorded current medication use as reported by the patient and caregiver during home visits, and changes in medication use in the weeks prior to the start of (sub) acute symptoms. The referral letter reported the medical history.

Psychiatric diagnoses were made according to DSM-IV-TR criteria. A probable (definite) diagnosis of delirium was made if all criteria for delirium were met. A possible diagnosis was made if all criteria but one were met.

### Statistical analysis

In phase I, we calculated the sensitivity and specificity of the items from the pilot questionnaire. We wanted to identify items that had 100% sensitivity for probable delirium. Items scored as unclear or missing were entered in the calculation as “yes” to not rule out delirium in these instances and maintain high sensitivity. The proportion of unclear/missing data varied between 0% and 16%. When no individual item proved to be 100% sensitive, we then tested combinations of the most sensitive items.

As a consequence of this approach, specificity of the (combined) items was mostly low and many non-delirious participants were screen-positive. We then decided to test two additional items, which are part of DRS-R-98, and caregivers can probably report, too, the presence of hallucinations (item 2) and suspicious delusions (item 3). For the same reason we used information reported in the referral letter: referred for emergency visit, prior delirium, prior dementia diagnosis, and hospital admission in the 3 months before referral. Unclear and missing data for the additional items were entered in the calculation as “no” to not rule out non-delirium cases and enhance specificity. In this way, we composed the short final DCQ.

In phase II, we calculated the sensitivity and specificity of the final DCQ. The proportion of unclear and missing data was 0% for node 1 (item 1), 13% for node 2 (item 2 and 3), and 34% for node 3 (item 4 to 7). Most missings on node 3 were related to missings on node 2 (95%). These items were considered as “yes” to not rule out delirium and obtain high sensitivity. For the same reason, probable and possible delirium were considered a positive diagnosis. We used the command “diagti” in Stata 14 (StataCorp, USA) to calculate test accuracy parameters (Seed and Tobias, 2001).

## Results

### Development of DCQ

Phase I was performed between January 2013 and July 2014 among patients referred to our memory clinic. We approached 162 caregivers and 112 consented to participate, and 64% of them had already been informed of the diagnosis. The remaining caregivers were not approached due to logistic problems initially and drop-out of one of the interviewers later on.

Of the 112 patients, 107 received a diagnosis and formed our study sample. Twenty-four were found to have probable delirium (percentage at intake 22.4%). They were 82.0 years (SD 9.7) on average at the time of referral, and 67.0% were female. Five patients had been referred for an emergency visit (usually performed the same day or the day after), and four of those had delirium. Referral for emergency intervention thus indicated an 80.0% *a priori* risk of delirium. As patients referred for an emergency visit do not need further triage to establish if they need to be assessed quickly, they were not included in the further analysis of test accuracy.

Table 1 shows the sensitivity and specificity of individual items of the pilot questionnaire. Sensitivity was highest (85%) for item 7 (patient is sometimes like before), and also high (80%) for items 4a (exact date of onset), 14 (sleeping disorders), and 17 (caregiver burden). The combination of items 7 (fluctuation) and 14 (sleeping disorders) had 100% sensitivity but specificity of 31%. In patients who tested positive on one of these items, a history of delirium had the highest specificity (99%), but other information had a high specificity (≥85%) as well. We found that the combination of DRS-R-98 item 2 (hallucinations), DRS-R-98 item 3 (delusions), a history of delirium, and recent hospitalization had good specificity (80%) and sensitivity (77%).

**Table 1.** Test accuracy of the pilot questionnaire items and other information^a^

	sensitivity (95% ci)	specificity (95% ci)
Items of pilot questionnaire		
1. Were you shocked by current behavior of pt?	50.0 (27.2–72.8)	45.1 (34.1–56.5)
2. Since when do you spend more time on pt?	75.0 (50.9–91.3)	39.0 (28.4–50.4)
3. Did the symptoms develop in a short time?	40.0 (19.1–63.9)	78.0 (67.5–86.4)
4a. Can you tell the exact day/week of onset?	80.0 (56.3–94.3)	43.9 (33.0–55.3)
4b. Duration of symptoms <6 months?	55.0 (31.5–76.9)	72.0 (60.9–81.3)
5. Does symptom severity fluctuate in a day?	70.0 (45.7–88.1)	46.3 (35.3–57.7)
6. Is pt sometimes less alert?	75.0 (50.9–91.3)	35.4 (25.1–46.7)
7. Is pt sometimes like you knew him/her before?	85.1 (62.1–96.8)	45.1 (34.1–56.5)
8. Is the behavior of the pt fickle?	75.0 (50.9–91.3)	42.7 (31.8–54.1)
9. Does pt tell stories you cannot follow?	55.0 (31.5–76.9)	65.9 (54.6–76.0)
10. Is pt sometimes restless or more physically active than normal?	40.0 (19.1–63.9)	64.6 (53.3–74.9)
11. Or is (s)he calmer or more inactive?	45.0 (23.1–68.5)	59.8 (48.3–70.4)
12. Does pt destroy or throw away valuable goods?	15.0 (3.21–37.9)	82.9 (73.0–90.3)
13. Does pt act more chaotic?	60.0 (36.1–80.9)	47.6 (36.4–58.9)
14. Has the sleeping pattern changed?	80.0 (56.3–94.3)	42.7 (31.8–54.1)
15. Did the physical health change?	55.0 (31.5–76.9)	50.0 (38.7–61.3)
16. Did the use of medication change?	65.0 (40.8–84.6)	58.5 (47.1–69.3)
17. Can you handle the current situation much longer? (no = screen-positive)	80.0 (56.3–94.3)	25.6 (16.6–36.4)
Combined items		
Item 7 or 4 positive	95 (75.1–99.9)	24.4 (15.6–35.1)
Item 7 or 14 positive	100 (83.2–100)	20.7 (12.6–31.1)
Item 7 or 17 positive	95.0 (75.1–99.9)	17.1 (9.7–27.0)
Other information available at referral		
DRS-R-98 item 2 hallucinations	35.0 (15.4–59.2)	90.8 (81.0–96.5)
DRS-R-98 item 3 delusions	50.0 (27.2–72.8)	84.6 (73.5–92.4)
History of dementia	15.0 (3.2–37.9)	89.2 (79.1–95.6)
History of delirium	15.0 (3.2–37.9)	98.5 (91.7–100)
Recent hospitalization^b^	25.0 (8.7–49.1)	95.4 (87.1–99.0)
Combined items		
DRS item 2 or DRS item 3 positive	60.0 (36.1–80.9)	83.1 (71.7–91.2)
History of delirium or recent hospitalization positive	35.0 (15.4–59.2)	93.8 (88.0–98.7)
DRS item 2, DRS item 3, history of delirium or recent hospitalization positive	80.0 (56.3–94.3)	76.9 (64.8–86.5)

Pt, patient.

a *n* = 102.

b Within 3 months.

Therefore, we composed the final DCQ as follows (see Dutch version in the appendix published as supplementary material):

- 1: Is the patient referred for an emergency visit?
- If true, there is a high risk of delirium. Further triage not required.
- If untrue, ask questions 2 and 3:
- 2: Did the sleeping pattern of the patient change since the onset of symptoms (that led to referral)?
- 3: Is the patient sometimes like before the onset of symptoms (that lead to referral)?
- If both questions 2 and 3 are untrue, further triage is not required.
- If question 2 or 3 is true, ask questions 4, 5, and 6:
- 4: Does the patient see or hear things that are not there?
- 5: Is the patient suspicious?
- 6: Has the patient ever had a delirium before?
- 7: Has the patient been recently hospitalized?

If question 4, 5, 6, or 7 is positive, a high risk of delirium exists and an emergency visit is required.

### Test accuracy

In phase II we tested the final DCQ in daily practice. This phase took place from November 2015 to June 2016. The questionnaire was to be implemented at two memory clinics of our mental health institution in the north and south of Rotterdam, the Netherlands. Unfortunately, the latter clinic had just changed the referral procedure in such a way that it was not possible to have a nurse or doctor administer the DCQ to a patient’s caregiver before the first visit. Therefore, the results below are based on the experience of 25 nurses and doctors with DCQ in the clinic in Rotterdam North, of which 7 administered 171 of 234 questionnaires (73%).

The psychiatric nurse to whom a patient was assigned administered DCQ to a patient’s caregiver by telephone before setting the date for the first visit. They could administer DCQ within 2 min. During psychiatric workup, 49 patients were found to have probable or possible delirium (21%). The mean number of days between referral and first visit dropped from 31.6 (SD 28.6) to 11.2 (SD 13.1) in patients with delirium after the introduction of DCQ (*p* = 0.001).

Of the 234 patients, 35 were referred for an emergency visit and 19 were found to have delirium. In the other 199 patients, DCQ indicated that 50 required an expedited visit and 30 had delirium. Sensitivity of the final DCQ was 73.5% (95% CI: 58.9–85.1%) and specificity 73.5% (95% CI: 66.5–79.7%). Positive predictive value was 42.4% (95% CI: 35.5–49.7%) and negative predictive value 91.2% (95% CI: 86.6–94.4%).

### Post hoc analyses

With the final 7-item DCQ, 13 of 49 patients with delirium were ruled out for expedited visit because they scored negative on the first 3 items (10) or on the last 4 items (3). Therefore, in a *post hoc* analysis, we tested the diagnostic accuracy of just the first three items (emergency visit; sleeping disorders; sometimes lucid as before). Sensitivity increased to 79.6% (95% CI: 65.7–89.8%) and specificity decreased to 70.8% (95% CI: 63.7–77.2%).

In addition, we checked the information gathered at intake about sleeping disorders (second item) and fluctuations (third item). Six of 10 patients scoring negative for these symptoms on DCQ showed to have one or both symptoms during intake. Sensitivity with the three-item DCQ incorporating this information was 85.7% (95% CI: 72.8–94.1%) and specificity 66.5% (95% CI: 59.2–73.2%).

## Discussion

In this study, we developed a short delirium caregiver questionnaire (DCQ) for triage and expedited the detection of delirium in older outpatients referred for cognitive screening. It consisted of seven items and can be administered within 2 min. Sensitivity was 73.5% and specificity 73.5%. An even shorter three-item DCQ turned out to perform better with sensitivity of 79.6% and specificity of 70.8%, if sleeping disorders and fluctuations had been reported for all patients who had these symptoms. Mean time to first visit in delirious patients was reduced from 31.6 to 11.2 days after the introduction of DCQ.

### Delirium screening instruments

We developed DCQ for triage and screening in older outpatients with cognitive impairment to improve timely recognition of delirium. Multiple screening instruments for delirium are available (Quispel-Aggenbach *et al*., 2018; Wong *et al*., 2005), but these are not suitable for the purpose of triage in outpatients. They are either not quick or require bedside assessment of the patient. In addition, the Informant Assessment of Geriatric Delirium (I-AGeD), which is also based on caregiver information, and DOSS were not sensitive in patients with dementia (Rhodius-Meester *et al*., 2013; Teale *et al*., 2018). Diagnostic instruments such as CAM and DRS-R-98 are not suitable for triage or screening either, because they are based on a full psychiatric assessment (Inouye *et al*., 1990; Trzepacz *et al*., 2001).

Furthermore, most screening and diagnostic instruments have been developed to maximize the percentage of patients correctly identified (whether with or without delirium). As a result, sensitivity and specificity are equally weighted. In our view, delirium screening and triage instruments need to be as sensitive as possible. Half of delirium cases are missed in hospitalized and institutionalized patients (De Lange *et al*., 2013; Siddiqi *et al*., 2006). This can be averted with an instrument that picks up as many cases as possible.

Our aim was to develop a questionnaire with very high sensitivity based on information that caregivers can provide (>90%). Two items of the seven-item DCQ originated from the pilot questionnaire and were included to optimize sensitivity, the other five to enhance specificity. Nevertheless, although sensitivity was good at 73.5%, it was not very high. Most false-negative delirious patients were not identified for expedited assessment (because the caregivers did not report sleeping disorders or symptom fluctuation at the time of triage even though these symptoms were found to be present during the psychiatric workup (62%)).

DCQ has been tested by psychiatric nurses but is likely to be user-friendly for other nurses, nurse assistants, psychologists, and physicians as well. In addition, it may be valuable in hospitalized and nursing home patients with cognitive impairment too. In those settings, the equivalent of an emergency referral in outpatients (item 1) would be the request for (quick) consultation by a psychiatrist or geriatrician and admission to the emergency department.

### Strengths and limitations

Strength of our study is that we used one large sample for the development of DCQ and another independent sample to test its accuracy and effect on time to first visit. With 234 patients and 49 cases of probable and possible cases of delirium (21%), the result of a *post hoc* sample size calculation indicated that our sample was large enough to detect a sensitivity of at least 90% (lower limit of confidence interval) (Jones, 2003). A limitation of our study was that we did not investigate the inter-rater or test-retest reliability of our triage tool.

Our instrument needs further development. It had a high sensitivity in the test sample, but somewhat less in the validation cohort. Perhaps, the questionnaire needs to be accompanied by an instruction on how to deal with unclear answers and caregivers not being able to provide answers, as well as an explanation of hallucinations and delusions for caregivers. The pattern of missings in the final DCQ also suggests that it might be better to remove the open question (to elicit a spontaneous account of symptoms) between items 1 and 2, and to administer all items to all caregivers. Alternatively, the sensitivity of sleeping disorders and symptom fluctuation of the pilot DCQ might have been overestimated because the instrument was administered to 65% of caregivers when delirium had already been diagnosed.

## Conclusion

DCQ appears to offer a seven-item instrument that is rapid and easy to use, and helps identify delirium quickly in outpatients referred for the assessment of cognitive impairment. The instrument needs further testing not just in outpatients but also in hospitalized and institutionalized patients.

## Supplementary material

For supplementary material accompanying this paper visit https://doi.org/10.1017/S1041610219001595. click here to view supplementary material
