# Supplementary material for: A short delirium caregiver questionnaire for triage of elderly outpatients with cognitive impairment: a development and test accuracy study
Source: Int Psychogeriatr. 2019 Oct 29;33(1):31–7. doi: 10.1017/S1041610219001595 (PMC8482371; doi:10.1017/S1041610219001595)
Supplement: Supplementary file 1 [file S1041610219001595sup001.docx]

Online Appendix A1

**Delirium Caregiver Questionnaire versie ingekort 1.2**

**H.J. Luijendijk & A.J.M. Stroomer-van Wijk & D.W.P. Quispel-Aggenbach**

**Algemene gegevens:**

Initialen en achternaam patiënt: …………………………….........................................................................

Geboortedatum:……………….................................................................

Geïnterviewde (onderstrepen): mantelzorger of verzorgende of anders

Datum afname:........................................................................................

Of: reden om niet af te nemen:……………………………………………...

1) Crisisaanmelding/ spoedbezoek gevraagd?* **Indien 1 ja**

**Indien 1 nee:**

Vraag waarom de patiënt verwezen is, als de geïnterviewde dat niet spontaan vertelt.

Klachten: ..................

2) Is het slaappatroon van uw ...... ook veranderd? Ja/ nee

3) Is uw .... soms weer even gewoon, zoals u hem/ haar vroeger kende? Ja/nee

**Indien 2 en 3 nee**

**Indien 2 en/of 3 ja:**

4) Ziet of hoort uw..... dingen die er niet zijn?*

5) Is uw ...... achterdochtig?*

6) Heeft uw .... wel eens een delier gehad?*

7)Is uw .... recent opgenomen geweest in een ziekenhuis?*

**Indien 4, 5, 6 en 7 nee Indien 4, 5, 6 en/of 7 ja**

**Risico op delier is laag (< 6%)**

- Reguliere intake plannen

**Risico op delier is hoog (> 70%)**

- Intake binnen 2 werkdagen

*Hoeft niet gevraagd te worden als dit al uit de aanmeldgegevens blijkt
